# The challenge and potential of photosynthesis: unique considerations for metabolic flux measurements in photosynthetic microorganisms

**DOI:** 10.1007/s10529-018-2622-4

**Published:** 2018-11-14

**Authors:** Cara L. Sake, Alexander J. Metcalf, Nanette R. Boyle

**Affiliations:** Colorado School of Mines, Golden, CO USA

**Keywords:** Metabolism, Isotopic labeling, Metabolic engineering, Photoautotrophic, ^13^C-MFA, Metabolites, Metabolomics, Carbon labeling, Cyanobacteria, Algae, INST-MFA, Quenching

## Abstract

Photosynthetic microorganisms have the potential for sustainable production of chemical feedstocks and products but have had limited success due to a lack of tools and deeper understanding of metabolic pathway regulation. The application of instationary metabolic flux analysis (INST-MFA) to photosynthetic microorganisms has allowed researchers to quantify fluxes and identify bottlenecks and metabolic inefficiencies to improve strain performance or gain insight into cellular physiology. Additionally, flux measurements can also highlight deviations between measured and predicted fluxes, revealing weaknesses in metabolic models and highlighting areas where a lack of understanding still exists. In this review, we outline the experimental steps necessary to successfully perform photosynthetic flux experiments and analysis. We also discuss the challenges unique to photosynthetic microorganisms and how to account for them, including: light supply, quenching, concentration, extraction, analysis, and flux calculation. We hope that this will enable a larger number of researchers to successfully apply isotope assisted metabolic flux analysis (^13^C-MFA) to their favorite photosynthetic organism.

## Introduction

**Fig. 1:**
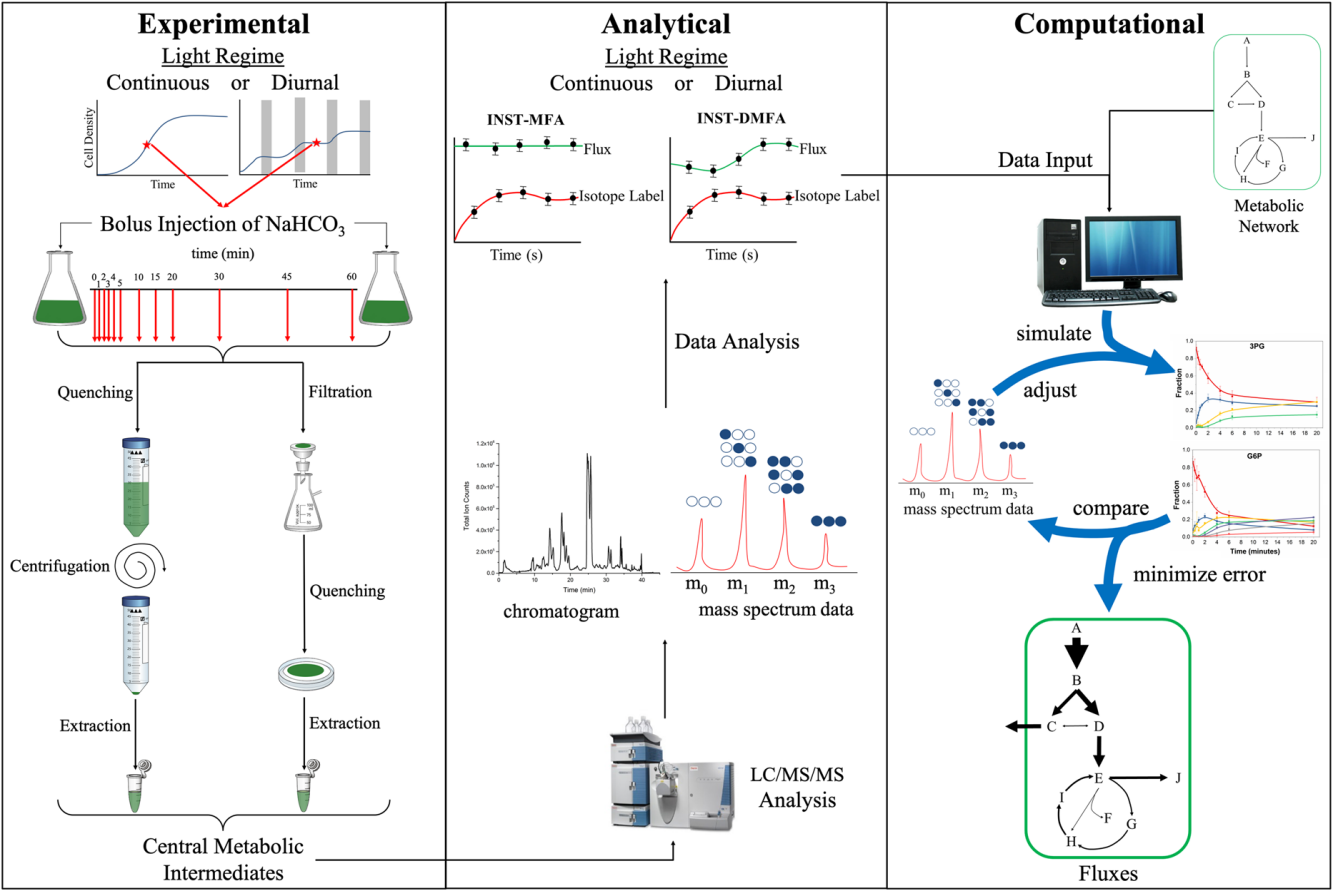
Workflow for the experimental, analytical, and computational aspects of flux measurements in photosynthetic organisms. To study photoautotrophic metabolic fluxes, transient experiments must be performed. After a bolus injection of ^13^CO_2_ (or more practically, NaH^13^CO_3_), cells must be rapidly sampled, quenched, and extracted. Concentrated metabolite solutions are then analyzed with LC/MS/MS, to produce a time series of mass distributions for each metabolite. This data is then incorporated into the model where adjustable parameters are iteratively changed until the predicted fluxes produce simulated data that minimizes errors when compared to experimental data

Due to their ability to convert carbon dioxide directly into desired products, metabolically engineered photosynthetic microorganisms, such as cyanobacteria and algae, have the potential to serve as sustainable sources of fuels, chemicals, and animal feed. Although this direct conversion is an attractive quality because it decouples the production of bioproducts from the food and feed supply, metabolically engineered algae and cyanobacteria have not yet had a significant commercial success like the production of 1,3-propanediol (Kurian 2005) or 1,4-butanediol (Yim et al. 2011) in *Escherichia coli*. The latter example actually had a relatively fast design to production phase, approximately 27 months (www.genomatica.com), due in part, to the use of metabolic modeling and isotope assisted flux analysis. Isotope assisted metabolic flux analysis, or ^13^C-MFA (Wiechert 2001; Yang et al. 2002; Young 2014b; Young et al. 2008, 2011a; Zamboni et al. 2009), provides an accurate snapshot of the metabolic fluxes in the cell at the time of sampling, allowing metabolic engineers to identify any bottlenecks or inefficiencies and design strategies to alleviate them. The use of isotope tracer experiments to measure metabolism dates back to the 1980s, and these early experiments laid the groundwork for modern ^13^C-MFA (Blum and Stein 1982). The first application of MFA based on stoichiometric modeling occurred in the 1990s as a direct precursor to modern ^13^C-MFA (Vallino and Stephanopoulos 2000). Early MFA relied on NMR technology, but the introduction of MS techniques in the early 2000s enabled labeling from intracellular metabolites and facilitated the study of more complex mathematical systems (Klapa et al. 1999). Despite the 30-plus years of development and progress, ^13^C-MFA was not expanded to the study of photoautotrophic metabolism until very recently. The main challenges in applying ^13^C-MFA to photoautotrophic metabolism are discussed further in this review, but they stem from the use of a single carbon substrate. To accommodate for the complexities of a single-carbon label, transient sampling methods were developed; they were coined isotopically instationary or nonstationary MFA (Noh et al. 2006; Noh and Wiechert 2006; Young et al. 2011b). Prior to the development of transient labelling techniques, photosynthetic organisms could only be studied in heterotrophic or mixotrophic conditions [*e.g.* (Xiong et al. 2010)]. (For a more detailed history of metabolic flux analysis and its role in metabolic engineering, the reader is directed to: (Villadsen et al. 2016; Woolston et al. 2013)). Recent advances in analytical capabilities and the availability of computational software packages have facilitated the wider implementation of ^13^C-MFA (Kajihata et al. 2014; Kogadeeva and Zamboni 2016; Young 2014a). Figure 1 provides a general overview of the workflow for the application of ^13^C-MFA to photoautotrophically grown microorganisms. While the implementation of any study to measure intracellular fluxes should be carefully designed, the unique characteristics of photosynthetic organisms and their metabolism requires special consideration. In this review, we will discuss the general workflow of a ^13^C-MFA experiment (further reading for specific applications that utilize the workflow shown in Fig. 1 include (Hendry et al. 2017b; Shastri and Morgan 2007; Wu et al. 2015)) and the unique challenges that must be considered when working with cyanobacteria and algae.

## Important experimental considerations for photosynthetic microorganisms

### Experimental design

Experimental design for photoautotrophic cells differs significantly from heterotrophic cells due in a large part to the uncoupling of carbon and energy substrates. Heterotrophic organisms use the same substrate for both carbon and energy; for example, when grown on glucose, microbes break down and build up molecules from the carbon backbone of glucose and oxidize carbon (from the same substrate) in the TCA cycle to provide reducing power and energy in the form of ATP. In contrast, photoautotrophs utilize a one-carbon substrate (CO_2_) as the building block for all carbon in the cell, and the light harvesting apparatus uses photons to regenerate reducing equivalents and ATP. The decoupling of carbon and energy metabolism introduces many complications in the design and implementation of experiments to measure fluxes; the discussion below will focus on how these are overcome.

#### Carbon metabolism

**Fig. 2:**
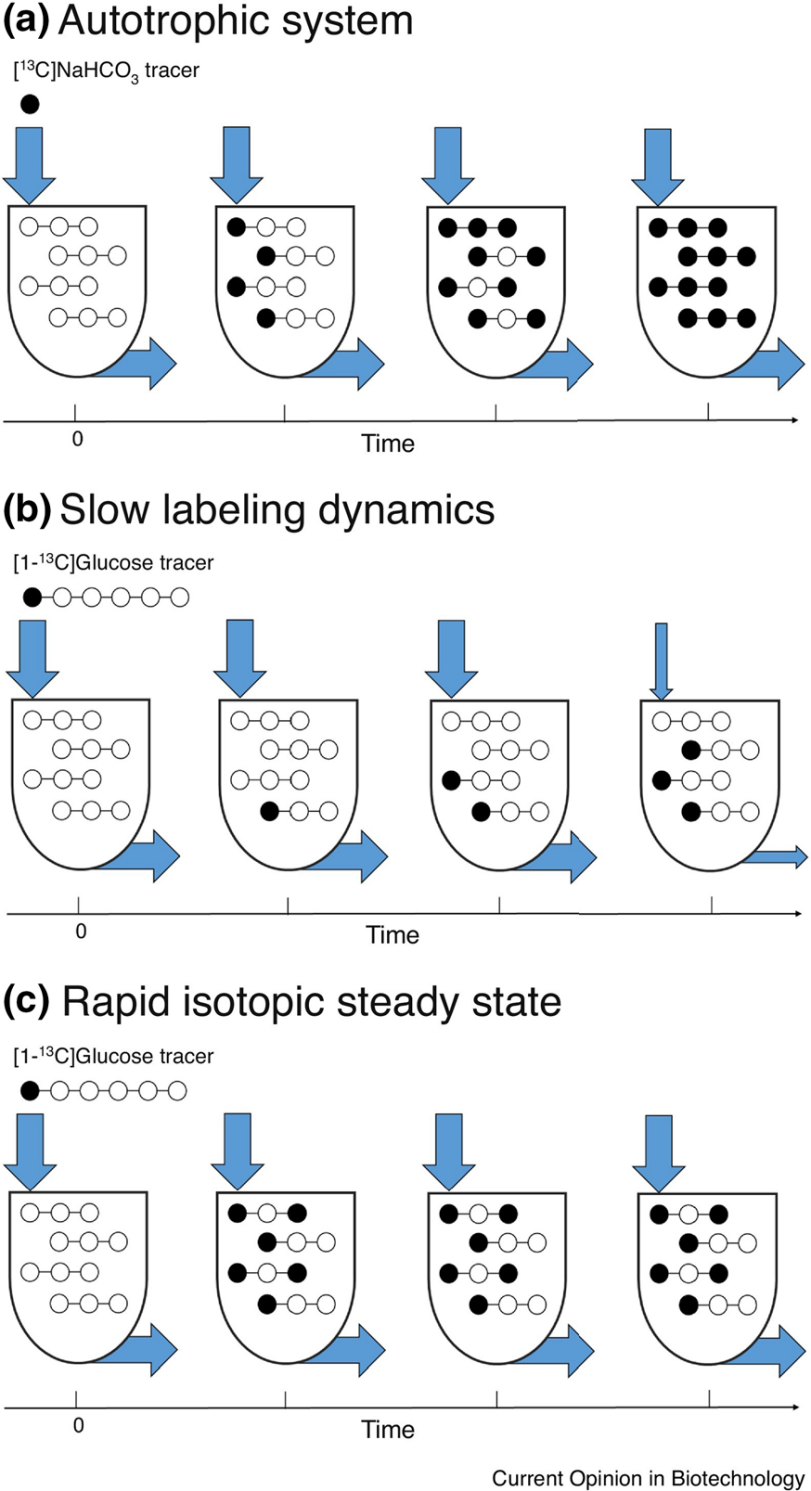
Applications of INST-MFA (cases **a, b,** and **c**) versus MFA (case **c** only). **a** Autotrophic systems. Due to the use of a single-carbon tracer, no unique flux solution can be calculated at isotopic steady state because all metabolites become uniformly labeled. **b** Slow labeling dynamics. The labeling of some metabolites may be too slow to achieve isotopic steady state within the timeframe that metabolic steady state can be maintained. Note that the fluxes (arrows) change before the labeling has fully equilibrated. **c** Rapid isotopic steady state. Although stationary MFA can be used to determine fluxes, INST-MFA can be used in some situations to improve estimates of exchange fluxes and pool sizes if rapid sampling is available. Arrows represent fluxes and tanks represent pool sizes at each time point. Figure used with permission from (Cheah and Young 2018a)

Experiments to measure heterotrophic carbon fluxes are performed when cells reach an isotopic and metabolic steady state. Metabolic steady state occurs when the metabolite pool sizes remain the same over the experimental time period and isotopic steady state occurs when the mass distribution of the isotopes remain the same. In heterotrophic organisms, the carbon substrate can be labelled on specific carbons to maximize the data gained from the experiment, however, if the substrate is a single carbon, at isotopic steady state everything in the cell would be labelled and no useful information would be gained by analyzing the isotope distribution (see Fig. 2 and (Cheah and Young 2018b) for an excellent discussion on this). Therefore, to collect data that can actually be used to calculate fluxes, labelling experiments must be performed transiently. Young et al. were the first to use this method to measure fluxes for autotrophically-grown cells (Young et al. 2011a). Their groundbreaking work illustrated that fluxes can be calculated from transient labelling data and that the majority of central metabolites have the most dramatic shifts in labelling in the first 2 min after the introduction of the label. This also highlights the need to develop rapid sampling because several samples must be taken in those first 2 min. Another challenge when designing experiments to measure autotrophic carbon fluxes is what label to use at night. For cells grown in diurnal light, carbon stored during the day, such as glycogen or starch, is used to maintain the cell and in many cases, perform cell division (Lena and Hirschie 2001; Sweeney and Beatriz 1989; Sweeney and Hastings 1958). Therefore, to track fluxes at night, a different label must be used, such as ^18^O_2_ or a ^15^N-labelled nitrogen source, but these experiments limit the type of information that can be gathered and again need to be performed transiently because they are single labelled atom substrates.

#### Energy

Light provides energy for the cell, so it is imperative that any experimental design maintains a consistent growth environment and provides light within the appropriate wavelengths: 440–700 nm, known as photosynthetically active radiation (PAR). Light intensity plays an important role in growth rate for photoautotrophs; increasing light intensity increases growth rate up to a saturation point, after which the cells suffer from photoinhibition (Long et al. 1994). Therefore, the choice of what light intensity to provide cells needs to be considered separately for each strain grown in the lab. On top of this, the consistency of the light in commercial incubators can be another important variable; in our experience, even incubators which are marketed specifically for growing photosynthetic organisms have a large variation in light intensity across the chamber. To minimize this effect, we recommend measuring PAR across the entire growth chamber and to only use the areas where light is most consistent. The shape of the growth vessel and depth of the culture is yet another consideration; larger volume culture flasks and bioreactors are prone to light gradients throughout the culture and cells deepest in the culture volume will have significantly less access to light than those on the culture’s edge due to self-shading effects. Ideally, light intensity would also be scaled with growth so on a per cell basis it is constant, but in practical terms this is very challenging to achieve. The choice of light source is also extremely important; the increased use of LEDs allow for greater control over the quality, quantity, and wavelength of light provided to cells (Schulze et al. 2014). Wavelength optimization is gaining more interest, with evidence that the spectral distribution of light significantly impacts culture behavior (Ooms et al. 2017). If spectral distribution is optimized or altered for a given application, it is important that it be kept constant during flux analysis experiments, to ensure that the culture remains at metabolic steady state. Light sources must also be regularly checked and replaced to ensure they are achieving desired light output. Overall, these variables associated with the choice of light source should be carefully considered, as light quality can impact cell size and pigment composition, changes that undoubtedly have metabolic consequences.

The choice of light conditions is another critical experimental parameter, as photosynthetic cells experience drastically different metabolism in continuous light versus diurnal light. Cells grown in continuous light are typically in metabolic steady state during the mid-exponential phase of growth, therefore the experiments are only transient in isotopic label. This represents all the published work to date measuring photoautotrophic metabolic fluxes because by providing continuous light, cells do not have to store carbon for maintenance at night and thus have much higher productivity. Unfortunately, this is not representative of natural conditions; the reason diurnal growth has not been studied using metabolic flux is that it introduces additional complexities. Due to the dynamic nature of diurnal growth, it is unlikely that the cell ever truly achieves a metabolic steady state, especially because cells have an extremely strong circadian clock that persists in gene expression even when grown in continuous light (de Winter et al. 2013, 2014). Although the experimental protocol to collect data is similar to isotopic non-steady state, the analysis of data from diurnal experiments is much more computationally intense, because both metabolic pool sizes and isotopic labels are changing. Despite these challenges, it is imperative that we develop methods that can measure diurnal fluxes so that we can develop strategies to engineer organisms grown in this environment.

### Quenching

Metabolism is an extremely rapid process, which can respond to environmental stimuli in fractions of seconds. In order to ensure that biological samples represent metabolism at the time of sampling, metabolism must be quenched immediately upon removal of the cells from the culture. The main method used to quench metabolism in photosynthetic microorganisms is to drop the temperature of the culture to 0 °C or less as rapidly as possible (Table 1); the approaches used to achieve this result vary and will be discussed below. The most widely used quenching method is cold (− 20 °C or below) methanol or methanol/water solutions. The prominence of this method probably stems from early quenching studies in both *E. coli* (Winder et al. 2008) and *S. cerevisiae* (Canelas et al. 2008; Koning and Dam 1992) which identified cold methanol quenching as the fastest, most efficient method to arrest metabolism without leakage of intracellular metabolites. Unfortunately, the results of the studies by Winder et al. (Winder et al. 2008) and Canelas et al. (Canelas et al. 2008) have been extrapolated to many other organisms without rigorous testing of how the quenching solution may or may not cause leakage. These studies found that 100% methanol at − 40 °C should be used for yeast and 60% methanol at − 48 °C should be used for *E. coli*. For the flux studies shown in Table 1, there is a large variation in methanol concentration and temperature for quenching. In general, eukaryotes can tolerate higher solvent and lower temperatures than prokaryotes, which require lower solvent concentrations and milder temperatures to prevent leakage from cold shock (Wittmann et al. 2004). The advantage of using a pure solvent or solvent/water solution is that cells can be kept at − 20 °C or below without the culture/quenching solution freezing. While cold methanol quenching is by far the preferred method due to ease of implementation, a few other approaches have also been applied to photosynthetic systems. This includes plunging the cell culture solution directly into liquid nitrogen and chilling to freezing or just slightly below using saline solutions. Due to the drastic temperature change when using liquid nitrogen and the culture solution freezing, this may cause leakage in prokaryotic organisms. Using temperatures closer to freezing implies that to achieve rapid arrest of metabolism, the ratio of quenching solution to cell culture must be higher and care must be taken to avoid the formation of ice. Unfortunately, it is very difficult to predict a priori what quenching method will minimize metabolite leakage; there are hypotheses that attribute the response to a combination of cell membrane/wall composition/structures, cell surface area, and cold shock (Zakhartsev et al. 2015), but these traits still do not lead to predictive responses. Therefore, quenching solutions should be evaluated for each new organism to which flux analysis is applied to minimize metabolite leakage.

**Table 1:** Selected examples of experimental protocols for flux measurements in photosynthetic organisms

Organism		Concentrating method (C)	Quenching method (Q)	Extraction method (E)	References	
Eukaryote	*Chlorella protothecoides*	Filtration	− 70 °C MeOH	40:40:20 CH_3_CN:MeOH:H_2_O	C	(Wu et al. 2014)
					Q	
					E	(Bennett et al. 2008)
	*Chlamydomonas reinhardtii* ^*^	Centrifugation	− 70 °C MeOH	Mechanical cell disruption with − 70 °C MeOH	C	(Boyle et al. 2017)
					Q	
					E	
	*Phaeodactylum tricornutum* ^*^	Centrifugation	Liquid nitrogen	Lyophilization followed by hydrolysis with HCl	C	(Zheng 2013)
					Q	
					E	
Prokaryote	*Synechocystis sp.* PCC 6803	Centrifugation	− 40 °C 60% MeOH	50% MeOH	C	(Young et al. Young et al. 2011a, b)
					Q	
					E	
	*Synechococcus elongatus* PCC 7942	Centrifugation	0 °C PBS	8:4:3 CHCl_3_:MeOH:H_2_O	C	(Jazmin et al. 2017)
					Q	
					E	(Folch et al. 1957)
		Centrifugation	− 5 °C minimal BG-11 medium	CHCl_3_:MeOH	C	(Abernathy et al. 2017)
					Q	
					E	(Ma et al. 2014)
	*Synechococcus elongatus* UTEX 2973					
	*Synechococcus* sp. PCC 7002	Centrifugation	− 20 °C 30% MeOH	− 20 °C 80% MeOH	C	(Qian et al. 2018)
					Q	
					E	
		Centrifugation	− 20 °C 60% MeOH	− 20 °C 80% MeOH	C	(Qian et al. 2017)
					Q	
					E	(Bennette et al. 2011)
		Filtration	− 20 °C 80% MeOH	− 20 °C 80% MeOH	C	(Bennette et al. 2011)
					Q	
					E	

*MeOH* Methanol, *PBS* phosphate buffered saline

*Heterotrophic experiment

### Harvesting/concentrating cells

To accurately measure intracellular fluxes, the cell must be removed from the spent growth medium prior to extracting intracellular metabolites; this step is often combined with cell concentration steps. When this step takes place in the sampling protocol can vary: if it is rapid, it can occur prior to quenching; otherwise it should be performed after quenching. Regardless of when this step is performed, it is important that the method chosen is sufficiently gentle to avoid cell damage and metabolite leakage. There are two main collection approaches: filtration and centrifugation (see Fig. 1). Filtration is typically performed prior to quenching; however, due to this, it must be done as quickly as possible to minimize changes in metabolism after sampling. This can be challenging because the light regime also needs to be maintained during filtration since the cells are still metabolically active. After filtration, cells must be separated from the filter, which can lead to loss of cells or increases in working volume of extraction solvent if the filter and cells are processed together. Centrifugation is typically performed after quenching; therefore care must be taken to keep the cells below 0 °C until metabolites are extracted. In practice, we have found this step is easier to implement, especially with rapid sampling, but the quenching solution must be carefully selected (and tested) to ensure no leakage of metabolites and no precipitation of medium components. Regardless of what cell concentration method is chosen, it is important to note that photoautotrophic growth typically results in much more dilute cell cultures than heterotrophic growth; therefore, the volume required is larger and sampling takes more time, either to draw out the sample with a pipette or to filter it. The choice of what cell concentration step to use depends heavily on the organism itself and what quenching approach is to be used.

### Metabolite extraction

After quenching and concentration, intracellular metabolites are extracted from the cell; depending on the extraction solution chosen, different groups of metabolites are extracted with different efficiencies (Rabinowitz and Kimball 2007). Historically, ^13^C-MFA has focused primarily on central metabolic intermediates, so that is what the discussion here will focus on; for specific classes of metabolites the interested reader is directed to Rabinowitz and Kimball (2007). Liquid/liquid extraction with organic solvents has been the preferred approach for microbes and organisms listed in Table 1. In general, it is easier to lyse the cell wall of prokaryotic organisms; therefore, cyanobacterial metabolite extractions tend to use milder solvents. Eukaryotic organisms have a more robust cell wall than prokaryotes; therefore, to achieve efficient metabolite extraction, they require the use of harsher solvents, mechanical disruption (such as french press or grinding), or freeze/thaw cycles to break open the cell wall. Ideally, metabolites would be specifically extracted from different cellular compartments (i.e. cytosol, mitochondria, plastid), however experimental options are extremely limited in this regard. Intracellular compartments are mechanically fragile and subcellular fractionation techniques are time intensive (Stitt et al. 1989); therefore it is extremely difficult both to isolate intact compartments and to do it at temperatures that prevent metabolic activity. There are approaches to account for compartmentation, such as considering compartment specific metabolites (Allen et al. 2007), but these approaches are typically used for steady state heterotrophic flux measurements. Due to the differences in cell wall composition between organisms, just as with the quenching step, the efficiency and efficacy of extraction must be examined for the specific organism being studied.

### Analytical chemistry

The choice of metabolite extraction method predetermines the general class of metabolites to be analyzed; therefore, since our previous discussion focused on central metabolic intermediates, we will again limit our discussion to these most critical metabolites. There are two main analytical approaches used for ^13^C-MFA: nuclear magnetic resonance (NMR) and chromatography coupled to mass spectrometry (MS). Each has its own distinct advantages and disadvantages. NMR has the advantage of being non-selective and non-destructive, meaning it is capable of detecting all metabolites in the sample simultaneously without altering the viability of the sample. NMR is also useful for determining the *position* of labeled carbon atoms and the structure of metabolites, which is useful for identifying unknowns. The main disadvantage of NMR is the low sensitivity and signal overlap among individual metabolites. While there have been some studies which use NMR, the majority of ^13^C-MFA experiments have utilized either gas-chromatography MS (GC/MS) or liquid chromatography MS (LC/MS or LC/MS/MS). Heterotrophic steady state ^13^C-MFA studies tend to favor GC/MS because they analyze mainly amino acids, carbohydrates and lipids. For isotopic nonstationary MFA (INST-MFA), LC/MS/MS is the method of choice. The type of data that is required from these experiments are the metabolite pool size (concentration), parent ion mass and fragment masses. Metabolite pool size measurements verify the assumption of metabolic steady state for INST-MFA; however they are not always directly required for flux determination and may instead be evaluated as adjustable parameters (Young et al. 2011a). Continuing advances in analytical chemistry have enabled identification of larger number of metabolites in a single run as well as higher resolution and faster acquisition rates in mass spectrometry; this may enable analysis of larger networks in the future instead of focusing on central metabolism alone.

## Important computational considerations for photosynthetic microorganisms

### Challenges

Extracting data from metabolic experiments and converting the information into measured fluxes is a computationally complex challenge. The first challenge is the inherent nonstationary behavior present in autotrophic models. As discussed above, photoautotrophic mass isotopomer distributions must be measured transiently, before isotopic steady state has been reached. The nonlinearity imposed by this consideration creates an “inverse problem”—the flux values must be calculated by fitting experimental data to a metabolic model, as they cannot be measured directly. Given the extensive parameter space, calculating fluxes for large networks can quickly become a computationally intense and challenging problem (Antoniewicz et al. 2006). However, there are several software packages designed to solve these problems without expert knowledge (Antoniewicz et al. 2007; Sriram et al. 2008; Sriram and Shanks 2004; Van Winden et al. 2002; Weitzel et al. 2013; Wiechert 2001; Young et al. 2008; Zamboni et al. 2009). One such package, INCA (Young 2014a), has been used to calculate isotopically nonstationary fluxes in plants (Ma et al. 2014), algae (Wu et al. 2014), and cyanobacteria (Abernathy et al. 2017; Hendry et al. 2017a; Xiong et al. 2015) and is perhaps the most widely used program for photoautotrophic fluxes.

Additionally, the high degree of compartmentalization in eukaryotic photoautotrophic cells can complicate accurate flux measurement. Some metabolites can be present in more than one compartment and may participate in different reactions within each separate compartment. It is possible to fractionate eukaryotic cells into compartments through non-aqueous fractionation (NAF) (Arrivault et al. 2014), and flux packages can process compartmental metabolomic data from NAF, making compartmental characterization feasible. However, even in experiments with the best possible outcome, the distribution is still a gradient and not a strict stepwise separation of intracellular organelles, complicating extraction and analysis (Dietz 2017). Moreover, NAF has been attempted but not achieved in algae due to the poor separation of intracellular compartments and metabolites (O’Grady 2013). NAF has the potential to improve compartmental analysis, but it not currently appropriate for use in photosynthetic microorganisms. Computational methods should be used to address compartmentalization instead.

Finally, photoautotrophic cells do not reach a metabolic steady state under diel light patterns. As the cell transitions between light and dark, different transcripts and enzymes activate and deactivate (Wagner et al. 2004; Zones et al. 2015). Shifts in cellular metabolism can even occur after days under constant light, due to persistent circadian rhythms (van Alphen and Hellingwerf 2015). In order to make sense of measured isotopic mass fractions under these changing conditions, dynamic MFA (DMFA) should be used, as DMFA assumes that metabolite fluxes and pool sizes are not held constant throughout the course of an experiment (Leighty and Antoniewicz 2011). This approach can generate fluxes and pools even as the metabolism shifts over time, making it invaluable for analyzing photoautotrophic cells as their metabolism shifts.

### Applications

It is worth noting that computational applications within metabolomics are not just limited to converting mass fractions into fluxes. Three areas of particular focus come to the fore. First, confidence and error calculations are quite important when assessing flux. Generally, error estimations are extracted from confidence values, and various methods exist to do so (Antoniewicz et al. 2006; Sokolenko et al. 2016). Fortunately, software tools such as INCA, Flux-P, and SUMOFLUX have built-in methods to calculate confidence bounds, sensitivities, and error from the data, giving the researcher a better understanding of reasonable values without excessive calculation (Benton et al. 1990; Ebert et al. 2012; Kogadeeva and Zamboni 2016; Young 2014a). Second, computational models can help reduce some complexities in mixotrophic or heterotrophic growth. When a cell grows mixotrophically, it consumes carbon dioxide and another reduced carbon source, such as glucose. Labeling only carbon dioxide leads to incomplete data, but labelling every carbon individually in the other source is expensive and inefficient. Software can be used instead to simulate the impact of specific labels on a given network of reactions, thereby guiding experimental design to maximize the impact of research funding and minimize the time spent on experiments (Young 2014a). Finally, software can also extend the value of flux measurements, as the measurements are used to fit and validate genome scale models. These models are useful for experimental design, as they can test knockouts and additions in silico, thereby providing guidance for potential genetic engineering approaches (Yim et al. 2011). These applications help demonstrate the importance of computational methods within photosynthetic metabolic engineering.

Experimental techniques and computational methods act in concert, and are matched in importance. Photoautotrophic flux measurement requires rapid and accurate sampling techniques, followed by powerful analysis. In order to produce high-quality, repeatable data, collection and analysis must be well practiced and documented. However, the effort required for generating this data is wasted without a thorough grounding in the computational complexities involved. An accurate picture of photoautotrophic metabolic fluxes can only be reached by combining both aspects.

## Conclusion

Since the first report of the use of instationary ^13^C-MFA to measure phototrophic fluxes in 2011, there have been a handful of studies on other organisms (Table 1). The increased complexity of experiments, analyses, and computations has limited the widespread use of this technology, but understanding how carbon is directed through metabolism is imperative if we aim to successfully engineer photocatalytic production strains for renewable chemicals, fuels, or pharmaceuticals. Here, we have presented the major steps in any ^13^C-MFA experiment and the major stumbling blocks in applying this technology to photosynthetic microorganisms. While challenges still exist within the field, there are promising avenues of research for each. For example, optimization of quenching and extraction protocols for specific species will reduce metabolite leakage and perhaps aide in the development of a predictive model based on cell wall structure/composition. Improvements in computational approaches will allow more complex analyses and result in better models of photosynthetic metabolism. Finally, improved models will allow for better experimental design, as research simulations will indicate where data collection will have the largest impact. The field of photosynthetic flux measurement is rapidly developing and will continue to grow in importance as usage of photosynthetic organisms increases.
